# Oxford Screening CSF and Respiratory samples (‘OSCAR’): results of a pilot study to screen clinical samples from a diagnostic microbiology laboratory for viruses using Illumina next generation sequencing

**DOI:** 10.1186/s13104-018-3234-8

**Published:** 2018-02-09

**Authors:** Colin Sharp, Tanya Golubchik, William F. Gregory, Anna L. McNaughton, Nicholas Gow, Mathyruban Selvaratnam, Alina Mirea, Dona Foster, Monique Andersson, Paul Klenerman, Katie Jeffery, Philippa C. Matthews

**Affiliations:** 1 The Roslin Institute, University of Edinburgh, Easter Bush, Midlothian, Edinburgh, EH25 9RG Scotland UK; 2 Edinburgh Genomics, Ashworth Laboratories, University of Edinburgh, Edinburgh, EH9 3FL Scotland UK; 3 The Wellcome Trust Centre for Human Genetics, Roosevelt Drive, Oxford, OX3 7BN UK; 4 Big Data Institute, University of Oxford, Old Road, Oxford, OX3 7FZ UK; 5 Nuffield Department of Medicine, Peter Medawar Building for Pathogen Research, University of Oxford, South Parks Road, Oxford, OX1 3SY UK; 6 Department of Infectious Diseases and Microbiology, Oxford University Hospitals NHS Foundation Trust, John Radcliffe Hospital, Headley Way, Headington, Oxford, OX3 9DU UK; 7 NIHR Biomedical Research Centre, University of Oxford, John Radcliffe Hospital, Headley Way, Headington, Oxford, OX3 9DU UK

**Keywords:** Metagenomics, Next generation sequencing, Illumina, Diagnosis, Virome, Microbiome, Infection, Respiratory, CSF

## Abstract

**Objectives:**

There is increasing interest in the use of metagenomic (next generation sequencing, NGS) approaches for diagnosis of infection. We undertook a pilot study to screen samples submitted to a diagnostic microbiology laboratory in a UK teaching hospital using Illumina HiSeq. In the short-term, this small dataset provides insights into the virome of human respiratory and cerebrospinal fluid (CSF) samples. In the longer term, assimilating metagenomic data sets of this nature can inform optimization of laboratory and bioinformatic methods, and develop foundations for the interpretation of results in a clinical context. The project underpins a larger ongoing effort to develop NGS pipelines for diagnostic use.

**Data description:**

Our data comprise a complete metagenomic dataset from 20 independent samples (10 CSF and 10 respiratory) submitted to the clinical microbiology laboratory for a large UK teaching hospital (Oxford University Hospitals NHS Foundation Trust). Sequences have been uploaded to the European Nucleotide Archive and are also presented as Krona plots through which the data can be interactively visualized. In the longer term, further optimization is required to better define sensitivity and specificity of this approach to clinical samples.

## Objective

Next generation sequencing (NGS) is an attractive approach to diagnosis of infection, with the potential to offer a single diagnostic pipeline to identify viruses, bacteria and fungi from a range of clinical samples [1–4]. However, there are multiple challenges in implementing such systems, and ongoing efforts are required to develop in vitro methods for handling diverse types of clinical samples, evaluate and improve sensitivity, reduce the high burden of human reads, distinguish contaminants or commensals from pathogenic organisms, and optimise positive and negative controls.

In this small pilot study, we focused on the detection of viruses from cerebrospinal fluid (CSF) and respiratory samples submitted to a routine diagnostic microbiology laboratory in order to evaluate a methods protocol and to provide a preliminary dataset for analysis, with a view to optimizing our laboratory approach and providing a foundation for improving bioinformatic algorithms.

A summary of this work was presented at the UK National Federation of Infection Societies (FIS) meeting, Birmingham, November 2017 [5]. We have subsequently focused specific attention on analysis of human herpes virus 6 (HHV-6) reads from within these samples, as this provides an interesting example of an organism which is widespread, potentially pathogenic [6–8] but may also be a bystander in clinical samples [9]. These results and analysis are presented in a separate manuscript [10].

## Data description

### Sample cohort

We randomly selected 10 cerebrospinal fluid (CSF) and 10 respiratory samples (20 different patients represented). CSF samples were submitted to the clinical diagnostic laboratory at Oxford University Hospitals NHS Foundation Trust between November 2012 and May 2014, and respiratory samples between May and December 2014. Prior to use for this research, samples had undergone routine clinical laboratory testing and were then stored at − 80 °C.

### In vitro methods

A full description of laboratory methods is provided in linked data files (see ‘OSCAR protocol’ listed in Table 1). In brief, samples were filtered through 0.45 μm spin column filters (Merck Millipore) to remove large cellular debris and bacterial contaminants. To increase the relative amount of encapsidated viral to host nucleic acids in the sample, we pre-treated the sample with DNAse and RNAse. Nucleic acids were extracted using the QiaAmp MinElute Virus Spin Kit (Qiagen) and recovered in nuclease-free water. Reverse transcription was primed by random hexamer primers and performed using SuperScript III reagents. Sequence independent amplification of cDNA (and DNA also carried over during extraction) was carried out by an initial addition of random octamer containing primer sequences. Subsequent PCR was performed using a single primer amplification. Illumina Nextera XT libraries were made from amplified cDNAs according to the manufacturer’s protocol and sequenced on the HiSeq 4000 platform with 150-base paired end reads at the Centre for Genomic Research (CGR), University of Liverpool, UK.

**Table 1:** Overview of data files/data sets

Label	Name of data file/data set	File types (file extension)	Data repository and identifier (DOI or Accession Number)	License
OSCAR protocol (methods)	File name: OSCAR protocol Details: This contains the full laboratory and bioinformatics methods Data set: Oxford Screening of CSF and Respiratory Samples (‘OSCAR’): Supplementary resources for a project using next generation sequencing (NGS) for identification of viruses from clinical laboratory samples	Portable document format (.pdf)	Figshare https://dx.doi.org/10.6084/m9.figshare.5670007 (Data citation 1)	CC-BY 4.0
Benefits and challenges of meta-genomic sequencing	File name: OSCAR table benefits and challenges Details: This table summarises some of the benefits and challenges associated with the application of metagenomic approaches (next generation sequencing, NGS) to the clinical diagnosis of viral infection Data set: Oxford Screening of CSF and Respiratory Samples (‘OSCAR’): Supplementary resources for a project using next generation sequencing (NGS) for identification of viruses from clinical laboratory samples	Portable document format (.pdf)	Figshare https://dx.doi.org/10.6084/m9.figshare.5670007 (Data citation 1)	CC-BY 4.0
Metadata for 20 samples used for meta-genomic sequencing	File name: OSCAR 20 samples metadata Details: This table contains the anonymised demographic and clinical details of 20 clinical samples (10 respiratory and 10 CSF), from a UK hospital microbiology laboratory, which were processed using a metagenomic pipeline Data set: Oxford Screening of CSF and Respiratory Samples (‘OSCAR’): Supplementary resources for a project using next generation sequencing (NGS) for identification of viruses from clinical laboratory samples	This file is available in Microsoft excel (.xlsx) and as comma separated variables (.csv)	Figshare https://dx.doi.org/10.6084/m9.figshare.5670007 (Data citation 1)	CC-BY 4.0
Krona plots of meta-genomic data	Oxford Screening of CSF and Respiratory Samples (‘OSCAR’): interactive data visualisation using Krona to display results from a pilot project using next generation sequencing (NGS) for identification of viruses from clinical laboratory samples	Hypertext Markup Language (.html)	Figshare https://dx.doi.org/10.6084/m9.figshare.5712091 (Data citation 2)	CC-BY 4.0
Sequence data	HHV6 reads and metagenomic data from clinical samples	Metagenomic sequences: Fastq HHV-6 reads are available in BAM and Fastq format	European Molecular Biology Laboratory (EMBL); European Nucleotide Archive (primary Accession PRJEB22949) (Data citation 3). Individual accession numbers (Data citations 4–27): SAMEA104355484; SAMEA104355485; SAMEA104355486 ; SAMEA104355487; SAMEA104421606; SAMEA104421607; SAMEA104421608; SAMEA104421609; SAMEA104421610; SAMEA104421611; SAMEA104355484; SAMEA104421613; SAMEA104421614; SAMEA104421615; SAMEA104421616; SAMEA104421617 ; SAMEA104421618; SAMEA104421619; SAMEA104421620; SAMEA104421621; SAMEA104355485; SAMEA104355486; SAMEA104355487; SAMEA104421625	Open access in accordance with ENA policy (https://www.ebi.ac.uk/ena/standards-and-policies)

### Bioinformatic analysis

A full description of laboratory methods is provided in linked data files (OSCAR protocol; see description and link in Table 1). In brief, the raw FASTQ files were trimmed to remove adapter sequences and to remove low quality bases. After trimming, reads < 20 base pairs were removed. The remaining reads were classified using Kraken v0.10.5-beta [11] against a reference database comprising the human genome in combination with all RefSeq genomes for viruses, bacteria and archaea. Human-tagged reads were discarded and the remainder were taken forward for analysis.

We used Kaiju [12] to confirm that the Kraken analysis was complete, using the full Genbank non-redundant protein database for viruses, bacteria and archaea. Reads were assembled de novo using metaSPAdes v3.10 [13, 14]. Assembled contigs were classified with Kraken [11], and results were visualised with Krona [15].

## Limitations

This study was undertaken as a pilot exercise to underpin refinement of both laboratory methods and analysis of metagenomic data from clinical samples. On the grounds of cost, we were restricted to analysis of a small number of samples. We did not set out to derive definitive clinical diagnosis, and the data should not be used for this purpose. There are inherent difficulties with using residual clinical samples, including bias introduced into sample selection (e.g. samples from patients with a high pre-test probability of infection tend to be used up in primary clinical testing and not available for research). In archived samples, the quality of nucleic acid may deteriorate over time (this may be especially pertinent for RNA viruses).

Our methods did not include positive and negative controls. For this reason, it is difficult to assess the sensitivity with which we detected any specific virus; it is possible that the in vitro methods may have enriched or depleted particular organisms or groups of organisms. In future, positive controls can be added by spiking samples with an organism that we anticipate would not be present in human samples, or running a parallel multiplex control panel [16].

Future studies, and an accumulation of practical experience, will be required to increase the certainty with which results of NGS platforms can be interpreted. While we anticipate instances in which a specific organism can be identified from a metagenomic dataset as the cause of a clinical syndrome, there are many instances in which ambiguity may arise as a result of the difficulties in discriminating between pathogenic organisms and contaminants or bystanders. Detailed prospective studies enrolling large numbers of study subjects are the ultimate aspiration, with the aim of collecting high resolution data that include medical history, other laboratory results, imaging, treatment and follow-up data.

## Abbreviations

CSF: cerebrospinal fluid
DNA: deoxyribonucleic acid
ENA: European Nucleotide Archive
NGS: next generation sequencing
RNA: ribonucleic acid
